# Determination of Intrinsic Drug Dissolution and Solute Effective Transport Rate during Laminar Fluid Flow at Different Velocities

**DOI:** 10.3390/pharmaceutics13060835

**Published:** 2021-06-04

**Authors:** Sara B. E. Andersson, Göran Frenning, Göran Alderborn

**Affiliations:** Department of Pharmaceutical Biosciences, Uppsala University, Box 591, SE-751 24 Uppsala, Sweden; goran.frenning@farmbio.uu.se (G.F.); goran.alderborn@farmbio.uu.se (G.A.)

**Keywords:** computational fluid dynamics, dissolution, fluid flow, intrinsic dissolution rate, microscopy, single particle dissolution

## Abstract

The objective of this study was to determine the intrinsic drug dissolution rate (IDR) and the solute effective transport rate of some drugs, using a single particle dissolution technique, satisfying qualified dissolution conditions. The IDR of three poorly water-soluble compounds was measured in milli-Q water using four different fluid velocities. The enveloped surface area of the particles was calculated from the projected area and the perimeter of the particle observed in the microscope. Furthermore, computational fluid dynamics (CFD) simulations were used to theoretically investigate the flow conditions and dissolution rate, comparing box shaped particles and spherical particles with similar dimensions and surface area as the particles used the experiments. In this study, the IDR measurement of the single particles was determined within 5–60 min using particles with an initial projected area diameter ($D_{p}$) between 37.5–104.6 µm. The micropipette-assisted microscopy technique showed a good reproducibility between individual measurements, and the CFD simulations indicated a laminar flow around the particles at all flow velocities, even though there were evident differences in local particle dissolution rates. In conclusion, the IDR and solute effective transport rate were determined under well-defined fluid flow conditions. This type of approach can be used as a complementary approach to traditional dissolution studies to gain in-depth insights into the dissolution process of drug particles.

## 1. Introduction

Dissolution studies are frequently performed during the development of pharmaceutical products. Dissolution methods can be divided into two categories, namely, methods to study the dissolution of single substances and methods to study the dissolution of formulated products. The former methods are typically used during preformulation and the latter methods during formulation development and quality control, e.g., as a means to predict the in vivo product performance [1,2].

The rate of dissolution of a solid is dependent on the area of contact between solid and liquid, normally approximated by the surface area of the dispersed powder or of a compacted powder, and the hydrodynamics of the fluid. The flow of the fluid in the vicinity of the solid will affect the diffusion layer thickness and the rate of convective transport of the dissolved molecules away from the particle surface, factors which will control the amount of molecules that appear in solution over time [3,4]. 

In the context of characterizing the dissolution of pharmaceutical preparations, the amount of dissolved drug with time is of obvious prime concern. However, there has also been great interest in deriving indications of the dissolution properties that are material specific and independent of variations in particle dimensions. The most frequently used indicator seems to be a surface area normalized dissolution rate (SAND), also denoted as the intrinsic dissolution rate (IDR). The most frequent way of assessing an IDR has been to use a disc of compacted powder which is rotated in a fluid, i.e., a single specimen approach [5,6,7]. A rotating disc method typically, and theoretically incorrectly, uses the original disc’s surface area as a constant in the calculation of the IDR [8]. Since pharmaceutical solid preparations contain typically polydisperse powders, IDR methods based on multiparticulate dissolution have also been used, e.g., a powder method [9,10] and a suspension method [11]. When the dissolution process is studied by utilizing a large number of particles, the dissolution condition for each single particle is varying due to the variations in particle size and shape [6]. It is, for example, reported that the particle shape may affect the diffusion layer thickness which will subsequently affect the dissolution rate [12]. Moreover, in the multiparticulate methods, rough estimates of the contact area between solid and fluid were used [9]. Finally, the fluid flow condition around each single particle is not known.

In order to enable the calculation of an intrinsic dissolution rate as well as a solute transport rate in the dissolution rate-controlling liquid layer in the vicinity of the particle, dissolution studies should ideally be conducted during qualified conditions, i.e., the particle surface area is accurately determined in real-time and the flow of fluid around the dissolving particle is a laminar flow of defined velocity. There may be several applications of such quality data, for example, the development and evaluation of dissolution models and the prediction of the effects of variations in the composition of the solvent, and such studies can thus be used as a complementary approach to traditional dissolution studies. It is, for example, pointed out [13,14] that a key aspect in understanding and modeling the dissolution of a powder is the prediction of the molecular transport from a single particle. One possible approach by which such qualified conditions can be achieved is a single particle dissolution technique assisted by fluid flow simulation. 

The objective of this study was to determine the intrinsic drug dissolution rate and the solute effective transport rate of some drugs using a dissolution technique satisfying qualified dissolution conditions and allowing the use of unmanipulated particles. The dissolution process is monitored by optical microscopy, an approach to study the dissolution of single particles also used earlier [2,15,16,17]. Here, a micropipette-assisted microscopy technique is used, a method that was originally developed to study microgels in different solutions [18] and that has also been used to study bead-drug interactions [19]. Computational fluid dynamics (CFD) is used to investigate the hydrodynamic conditions around the single particle.

## 2. Materials and Methods

### 2.1. Materials 

Ibuprofen was provided from Orion (Espoo, Finland). Carbamazepine, indomethacin and dimethyl sulfoxide (DMSO) were purchased from Sigma-Aldrich (Steinheim, Germany). The DMSO was used to prepare a stock solution for establishing a standard curve in the disc IDR measurement. The medium used for dissolution measurements was milli-Q water (Millipore, Purelab Flex 2, Elga LabWater, Lane End, UK). The solubility in water and the physicochemical properties of the compounds are presented in Table 1. 

### 2.2. Apparent Particle Density

The apparent particle density (number of independent measurements, n = 3) for each compound (Table 1) was measured using helium pycnometry (AccuPyc II, Micromeritics, Norcross, GA, USA). For each measurement, a sample of 2–4 g was put into a sample holder with volume of 10 cm^3^ and the determined powder volume was an average of five consecutive measurements on each sample. 

### 2.3. Scanning Electron Microscopy

Scoop sampled particles were sprinkled over a double sided carbon tape adhered to a metal stub and coated with gold/palladium under argon (Polaron, QuorumTechnologies Ltd., Newhaven, UK). Particle images were thereafter taken by scanning electron microscopy (SEM, Leo (Zeiss) 1550 Schottky, Oberkochen, Germany) at six magnifications, 100×, 200×, 300×, 500×, 1000× and 5000×, using an accelerating voltage of 2.0 kV.

### 2.4. Single Particle Dissolution

#### 2.4.1. Preparation of Drug Slurry

An excess amount of each drug was stirred in milli-Q water at room temperature overnight to create the saturated solutions. Thereafter, the excess solid material was removed from the suspensions by filtration using a filter with a pore size of 0.2 µm. A slurry of each drug was subsequently prepared by adding approximately 2 mg of particles to 5 mL of the saturated solution, a small amount of which was introduced into a petri dish (1–2 drops from a transfer pipette). Using a micropipette, a single particle was drawn from the added particles which was used in the single particle dissolution experiment (see below). The procedure was repeated for all compounds used.

#### 2.4.2. The Micropipette-Assisted Microscopy Technique

A micropipette-assisted microscopy technique was used to study the dissolution rate of single particles [18]. The micropipettes used to draw particles from the slurry were prepared from G-1 glass capillaries. The G-1 glass capillaries were divided into two parts using a pipette puller PN-31 (Narishige, Tokyo, Japan). The edge of the capillaries were polished with a microgrinder EG-400 (Narishige, Tokyo, Japan) to create smooth ends with a diameter of approximately 50 µm. The micropipette was inserted into a microinjector (IM-11-2, Narishige, Tokyo, Japan), from which a suction was applied to keep the particle from detaching. An optical light microscope (Olympus Bx-51, Olympus, Tokyo, Japan) equipped with a DP digital camera (Olympus, Tokyo, Japan) was used to detect single particles of the size of approximately 100 µm in the petri dish. After sampling a single particle, the micropipette was used to turn the particle in different directions, making it possible to measure the three main dimensions of the particle, i.e., the breadth (B), the length (L) and the thickness (T).

**Figure 1:**
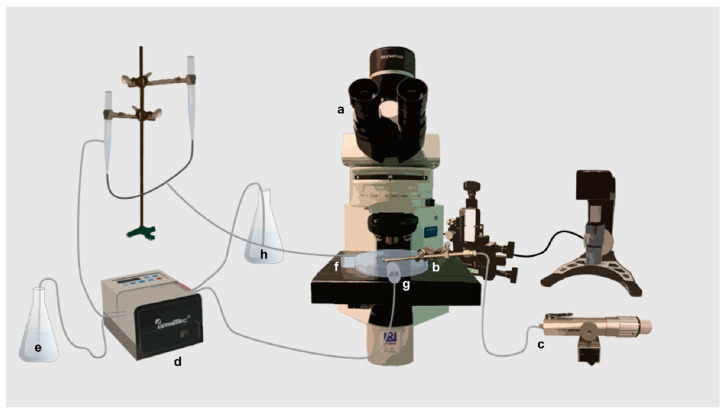
A schematic illustration of the micropipette-assisted microscopy technique. The optical light microscope (**a**) was used to detect single particles attached to a micropipette inserted into a microinjector (**b**) with an applied suction (**c**). A peristaltic pump (**d**) with a continuous fluid flow of medium (**e**) was connected to a flow-pipette (**f**). To obtain a constant fluid volume in the petri dish, a second flow-pipette (**g**) was connected to the peristaltic pump, removing fluid (**h**) at the same rate.

The micropipette, together with the attached single particle, was inserted into a flow-pipette, attached to the petri dish, and connected to a peristaltic pump (Pharmacia, Uppsala, Sweden) with a continuous fluid flow. The fluid flowed from the peristaltic pump, through the flow-pipette and out into the petri dish. To get a constant fluid volume in the petri dish a second flow-pipette was connected to the peristaltic pump, removing fluid at the same rate. A schematic drawing of the experiment set-up is presented in Figure 1.

The petri dish was filled with a saturated water solution of the compound from which the single particle was collected and placed into the flow-pipette. The saturated solution was used to adjust the fluid flow and once the dissolution experiment was commenced the saturated solution was switched to milli-Q water as the dissolution medium. The volume flow rate of the fluid was measured and the average fluid velocity ($\bar{v}$) was calculated from the measured volume flow rate using Equation (1).

(1) $$\bar{v}=\frac{Q/60}{\pi d^{2}/4}$$

where $\bar{v}$ is the average fluid velocity in the flow-pipette (mm/s), Q is the volume flow rate (mL/min) and d is the inner diameter (mm) of the flow-pipette. Assuming laminar flow, the fluid velocity profile is parabolic and the velocity in the center of the micropipette equals twice the average velocity [18]. 

To characterize the flow in the system, the Reynolds number (Re) [22] was calculated using Equation (2).

(2) $$Re=\frac{Ul}{v_{kin}}$$

where U is the flow speed, $v_{kin}$ is the kinematic viscosity of the fluid and l is the diameter of the tube or the diameter of a single particle.

The single particle dissolution experiments were run in triplicate and presented as mean values with standard deviations (SD). For each compound, particles with similar shape and size were selected for the measurements. In order to follow the dissolution process in real time, images of the particle were regularly taken using the optical light microscope (Olympus Bx-51, Olympus, Tokyo, Japan) and the DP digital camera (Olympus, Tokyo, Japan). The images captured during the dissolution experiment were analyzed by the camera software (Olympus cellSense Dimension, Tokyo, Japan).

### 2.5. Micromeritic Properties during Particle Dissolution

#### 2.5.1. Particle Dimensions

During the dissolution experiments, the particle was held in such a position that its length L and its thickness T were observed in the microscope. The particle was held in the same position during the whole dissolution process and the decrease of the third dimension (B) had hence to be calculated assuming that the third dimension decreased in parallel with the other two dimensions, i.e., the average decrease in percentage of the L and the T was used to calculate the decrease of the B. The projected area ($A_{p}$) of the particle observed in the microscope was determined and transformed into a projected area diameter ($D_{p}$) of the particle, i.e.,

(3) $$D_{p}=\sqrt{\frac{4A_{p}}{\pi }}$$

#### 2.5.2. Particle Surface Areas

The enveloped surface area of the particle (S) was calculated from the $A_{p}$ and the perimeter (P) of the particle observed in the microscope and the calculated particle B:

(4) $$S=2A_{p}+P\times B$$

Since a part of the enveloped area was occupied by the micropipette, and hence not in contact with water, the effective interfacial area ($S_{eff}$) was calculated by subtracting the cross sectional area of the micropipette tip from the enveloped particle surface area, as given in Equation (5):

(5) $$S_{eff}=2A_{p}+P\times B-\pi r^{2}$$

where r is the radius (µm) of the micropipette.

#### 2.5.3. Particle Shape Factors

As indications of particle shape, two shape factors based on dimensions and areas of the particles were calculated. Firstly, the flakiness (F) was calculated by dividing the particle length L with the particle thickness T, i.e.,

(6) $$F=\frac{L}{T}$$

Secondly the sphericity (Sp) was calculated as the ratio of the surface area of a sphere of equivalent volume ($S_{eq}$) as the studied particle and the enveloped surface area of that particle (S) [23], i.e.,:

(7) $$Sp=\frac{S_{eq}}{S}=\frac{\pi^{1/3}{\left(6V\right)}^{2/3}}{S}$$

#### 2.5.4. Particle Volume and Mass

**Figure 2:**
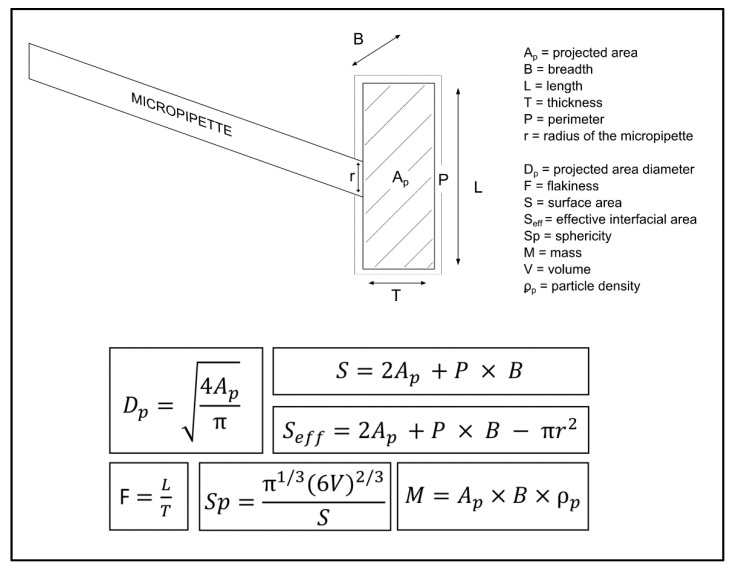
An illustration of a single particle attached to the micropipette. The known properties; $A_{p}$, B, L, T and P are used to calculated $D_{p}$, F, S, $S_{eff}$, Sp and M.

Since the particles studied were irregular, the volume V of a single particle was calculated as the product of its projected area ($A_{p}$), measured from the particle image, and its calculated breadth B, i.e., $A_{p}\times B$. The mass of the particle (M) was then calculated by Equation (8):

(8) $$M=A_{p}\times B\times \rho_{p}$$

where $\rho_{p}$ is the particle density. The micromeritic properties are explained in Figure 2.

### 2.6. Dissolution Rate Measurements

#### 2.6.1. Dissolution Rate of Single Particles

The IDR of a single particle was calculated in two ways. Firstly as the slope of a plot of the cumulative dissolved amount of compound per surface area (µg/cm^2^) against time (min) and secondly, as the ratio between change in particle weight and surface area at time ‘t’, i.e.,

(9) $$\mathrm{IDR}=\frac{W_{0}-W_{t}}{t\left(S_{0}-S_{t}\right)/2}$$

where $W_{0}$ is the initial weight of the particle, $W_{t}$ is the weight at time ‘t’, $S_{0}$ is the surface area of the initial particle and $S_{t}$ is the surface area at time ‘t’.

#### 2.6.2. Dissolution Rate of a Disc

For ibuprofen, an IDR was also determined by a rotating disc method using a µDISS Profiler (pION Inc., Billerica, MA, USA). A standard curve was established using a DMSO-stock, where 6 aliquots were added to 3 mL milli-Q water and stirred for 1 min at 800 rpm. Discs with a surface area of 0.707 cm^2^ were prepared by compressing approximately 5 mg of compound in a disc holder using a mini-IDR compression system (Heath Scientific, Milton Keynes, UK). The disc holder was then placed into a magnet stirrer, put into vials and 10 mL of milli-Q water was added at the same time as the experiment was commenced. The disc IDR measurement was run in triplicate, at room temperature, at 100 rpm.

### 2.7. Solubility Measurements from Powder Dissolution

Powder dissolution measurements were performed using the µDISS Profiler. The plateau of the dissolution curve was used to determine the solubility of the compounds. A standard curve was established using the same method as described in Section 2.6.2. Each compound was weighed into 20 mL vials, and magnetic cross stirrers were placed into the vials. The in situ UV probes were lowered into the vials and the measurement was commenced at the same time as 15 mL milli-Q water (room temperature) was added. The magnetic cross stirrers were immediately switched on (100 rpm). The concentration was determined using the in situ UV probes with a predefined time interval for up to 24 h. Since carbamazepine was challenging to measure in the µDISS Profiler, a solubility value determined by Sehic et al. was used [20].

### 2.8. Simulation Models for Single Particle Dissolution

**Figure 3:**
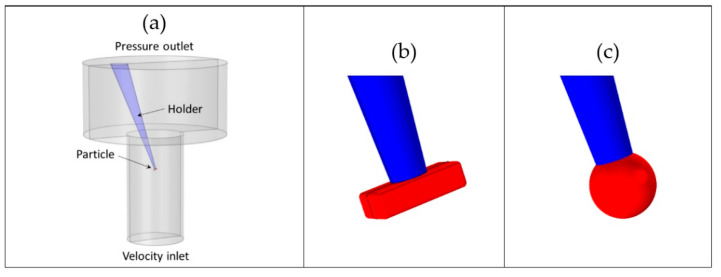
Geometry of (**a**) computational domain, (**b**) a rectangular particle and (**c**) an equivalent sphere. The particle is shown in red and the holder in blue.

In the CFD simulations, the dissolution medium was considered an incompressible linearly viscous (i.e., Newtonian) fluid and gravity was disregarded. Hence, momentum balance reduces to the incompressible Navier−Stokes equation,

(10) $$\rho_{f}\dot{v}=-\nabla p+\mu \nabla^{2}v$$

where $\dot{v}$ is the material time derivative and $\nabla^{2}v$ is the spatial Laplacian of the fluid velocity v and ∇p is the spatial pressure gradient. The fluid density $\rho_{f}$ and dynamic viscosity μ are constants (Table 2). Fick’s law, with a constant diffusion coefficient D (Table 2), was assumed applicable, as appropriate for dilute solutions. Hence, conservation of dissolved drug reduces to the diffusion equation,

(11) $$\dot{c}=D\nabla^{2}c$$

where $\dot{c}$ is the material time derivative of the drug concentration c. As illustrated in Figure 3a, a cylindrical domain with diameter 1.6 mm and length 3 mm was used to mimic the flow-pipette, with a fully developed (i.e., parabolic) inlet velocity profile prescribed at the lower end (average velocity $\bar{v}$ and maximum velocity $2\bar{v}$). The cylinder mimicking the flow-pipette emptied into a larger domain, also of a cylindrical shape (diameter 4 mm and length 2 mm), that was used to represent the ambient liquid. A pressure boundary condition was used on the top face, thus enabling outflow of liquid. An oblique conical holder kept a particle on the center axis of the flow-pipette, at a distance of 1 mm from its outflow end. The mirror symmetry of the system was used to reduce the size of the computational domain.

Rectangular particles of dimensions $110\times 50\times 30\;{\mu m}^{3}$, with rounded edges and corners (radius 5 μm), were considered, as illustrated in Figure 3b. For comparison, equivalent spherical particles of the same surface area were also used (diameter 76.5 μm), as shown in Figure 3c. Simulations were performed for a range of inlet flow velocities $\bar{v}$ (including the ones investigated experimentally) using COMSOL Multiphysics 5.4 (COMSOL AB, Stockholm, Sweden). Drug dissolution was studied by fixing the dissolved drug concentration at the value $c_{s}$ = 74.1 µg/mL = 74.1 g/m^3^ at the particle boundary (Table 2). 

From the simulations, the liquid velocity and drug concentration were determined. The overall drug dissolution rate ϕ was calculated as a surface integral of the flux out from the particle surface. For comparison, the dissolution rates were related to the ones corresponding to diffusional release from a spherical particle, i.e., the Sherwood number Sh was calculated as

(12) $$Sh=\frac{\phi }{\phi_{0}}$$

where

(13) $$\phi_{0}=4\pi R^{2}\times D\frac{c_{s}}{R}=4\pi RDc_{s}$$

is the release rate from a spherical particle of radius R in absence of convection.

## 3. Results and Discussion

### 3.1. Simulations of Fluid Flow and Dissolution of a Single Particle

Dissolution measurements are frequently performed during the development of new pharmaceutical products. In compendial basket and paddle apparatuses, such as the USP 1 and USP 2 methods, the agitation rate is typically specified in terms of the number of revolutions per minute (rpm) and it is hence difficult to estimate the local fluid flow pattern and velocity around a solid product or fragments thereof [24]. In a flow-through apparatus, such as the USP 4 method, the liquid flow velocity (mm/s) can be determined [25,26]. The advantage with a flow-through dissolution technique is that the sink condition is maintained during the experiment due to the constant introduction of new dissolution medium into the system [27]. 

The flow rates have been suggested to be set between 4 to 16 mL/min in the flow-through (USP 4) dissolution apparatus [26]. Depending on the diameter of the cell (12 mm or 22.6 mm) the corresponding average flow velocity values have been estimated [27] to be approximately 0.33 mm/s and 2.4 mm/s for 8 mL/min and 16 mL/min, respectively. Those fluid velocities are considerable lower than the fluid velocities reported for the paddle apparatus, the basket apparatus and the µDISS Profiler [27,28]. At 50 rpm, simulated velocity values range from zero to values up to 67 mm/s in the paddle apparatus and 26 mm/s in the basket apparatus [29]. Computational fluid dynamics (CFD) simulations assessing different agitator geometries and stirring rates in the µDISS Profiler show that the rotating disc is the least effective agitator with 17 mm/s per 100 rpm, whereas the cross stirrer is the most effective with an average flow velocity of 57 mm/s per 100 rpm [28]. 

The fluid velocities in the single particle dissolution experiments were calculated by Equation (1). Since the velocity in the center of the micropipette is estimated to be twice the average velocity [18], this was taken into consideration, and the fluid velocities in the vicinity of the particle were calculated to be 46, 66, 88 and 103 mm/s. These fluid velocities are hence similar to the velocities reported for the paddle apparatus, the basket apparatus and the µDISS Profiler, as discussed above. To confirm a laminar flow, the Reynolds number was calculated for the flow-pipette as well as the particles using Equation (2). It was calculated to be 21–46 for the flow-pipette, depending on fluid velocity (46–103 mm/s) and to be approximately 1–6 for the particles, depending on particle size and fluid velocity. Hence, the Reynolds numbers calculated are significantly lower than the cut-off value indicating a turbulent flow, which is estimated to be approximately 2300 for a tube [30]. 

**Figure 4:**
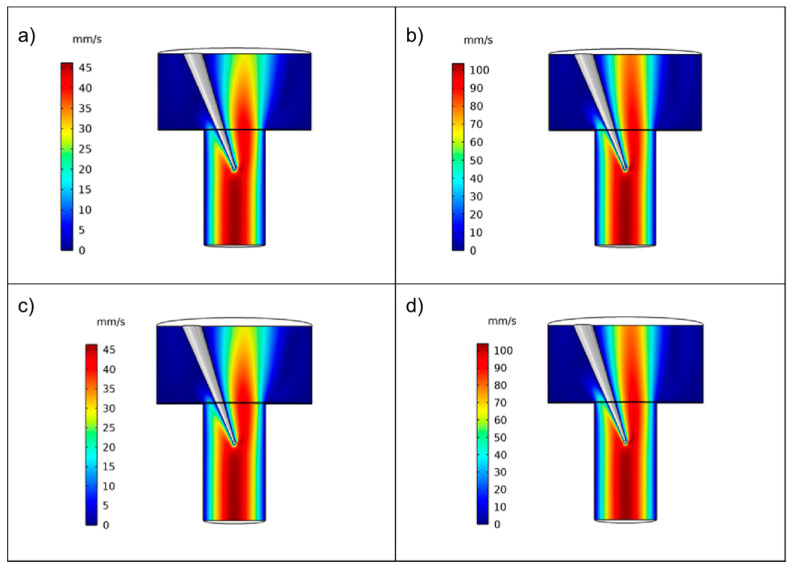
Magnitude of fluid velocity across the flow-pipette for a rectangular particle (**a**,**b**) and an equivalent sphere (**c**,**d**) for two different fluid velocities: (**a**,**c**) 46 mm/s and (**b**,**d**) 103 mm/s.

**Figure 5:**
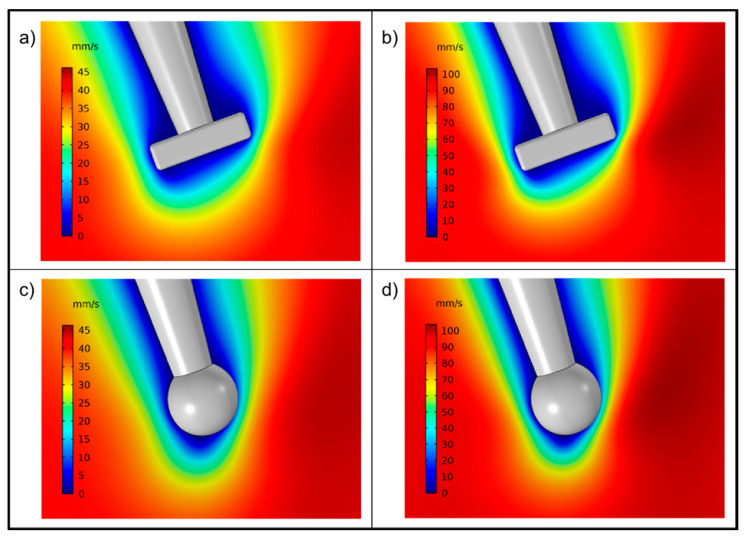
Magnitude of fluid velocity in the symmetry plane for a rectangular particle (**a**,**b**) and an equivalent sphere (**c**,**d**) for two different fluid velocities: (**a**,**c**) 46 mm/s and (**b**,**d**) 103 mm/s.

The fluid flow pattern in the experimental setup used in this work was assessed by CFD (Figure 4 and Figure 5). A gradient in flow velocity across the flow-pipette was obtained, i.e., a parabolic velocity profile which was similar for the two flow rates used in the simulation (46 mm/s and 103 mm/s). The continuous flow of fluid along the flow-pipette was disturbed around the micropipette and a marked gradient in flow velocity was obtained around the micropipette and the attached particle.

Simulations of the flow around the particle were done for two particle shapes, i.e., a sphere and a box, the latter mimicking the shape of the ibuprofen particles used as one of the model compounds in the dissolution experiments (see below), and magnified views of the fluid flow are provided in Figure 5. 

As expected, the finer details of the flow pattern were affected by the particle shape and the fluid velocity profile was more uniform for the sphere than for the box. However, the effect of particle geometry on the gross features of the liquid flow was small. Moreover, there were no indications of turbulent flow around either of the particles, i.e., a laminar flow around the particle was obtained.

**Figure 6:**
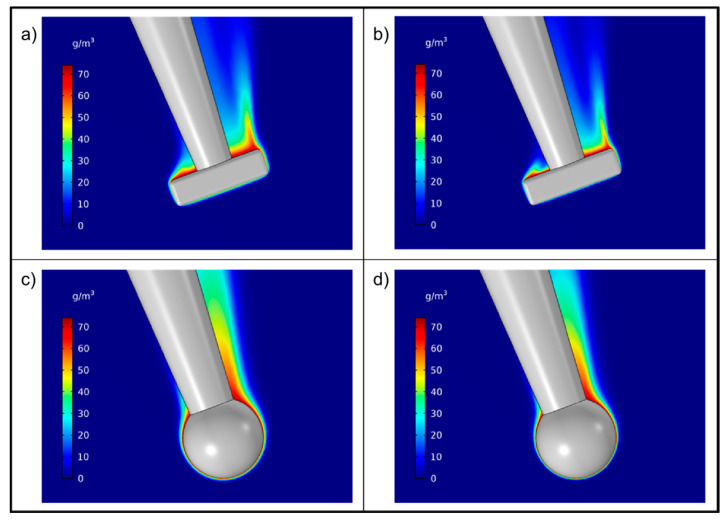
Drug concentration in the symmetry plane for a rectangular particle (**a**,**b**) and an equivalent sphere (**c**,**d**) for two different fluid velocities: (**a**,**c**) 46 mm/s and (**b**,**d**) 103 mm/s.

In Figure 6, the drug concentration (mol/m^3^) around the particle during dissolution, as obtained from simulations, is depicted in the symmetry plane. For the box particle, the layer of fluid containing an appreciable amount of dissolved drug is thin, even along the particle surface facing the incoming fluid flow and along the surfaces parallel to the fluid flow. For the particle surface on the reverse side to the surface facing the incoming flow, a thicker and more uneven drug layer was obtained with a spike at one box corner. For the sphere, a similar overall pattern was obtained but with a less heterogeneous drug layer on the reverse side of the sphere to the incoming flow.

**Figure 7:**
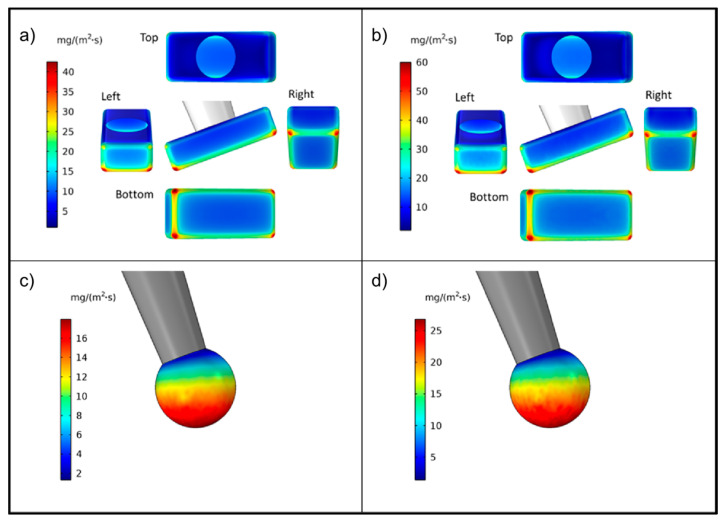
Dissolution rate from a rectangular particle (viewed from different angles) and an equivalent sphere for two different fluid velocities: (**a**,**c**) 46 mm/s and (**b**,**d**) 103 mm/s.

Consistent with these observations, there were evident differences in simulated dissolution rates on the different faces of the particles, as seen in Figure 7. For the box particle, the dissolution proceeded more rapidly at the edges and corners (Figure 7a,b). As a result, an initially box-shaped particle is expected to become somewhat rounder in shape as dissolution proceeds. Dissolution was most rapid on the faces directed towards the incoming flow, intermediate for faces perpendicular to the flow and smallest on faces that opposed the incoming flow. Specifically, the following approximate average dissolution rates were obtained for a fluid velocity of 46 mm/s (Figure 7a): left face: 13.0, bottom face: 11.0, side face (depicted in the middle of Figure 7a): 9.6, right face: 8.8 and finally top face: 2.5 mg/(m^2^s).

The rank order was the same for the higher fluid velocity. Taken together, these values indicate a similar rate of reduction in the length and breadth, which in turn is larger than the rate of reduction of the thickness. However, since the initial thickness was considerably smaller than the other main dimensions, the relative rate of change would be larger for the thickness than for the length and breadth (and of these, the relative change of the breadth would be somewhat larger than that of the length). For the equivalent sphere, a gradual reduction of the dissolution rate along the direction of the flow was obtained (Figure 7c,d).

**Figure 8:**
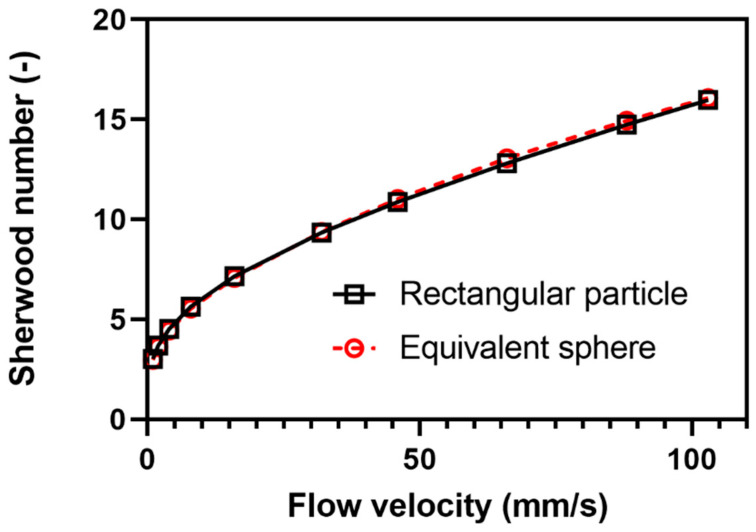
Overall dissolution rate (expressed in terms of the Sherwood number) for a rectangular particle and an equivalent sphere.

In Figure 8, the Sherwood number, i.e., an indication of the overall dissolution rate, is presented as a function of inflow rate of fluid for the two types of particles. The dissolution rate increased non-linearly with increased fluid flow rate for both particle shapes. The curve shape is consistent with prior results obtained for convective enhancements of heat and mass transfer as expressed, e.g., by the Ranz−Marshall correlation [31].

Despite the evident differences in local dissolution rates observed between the box particle and the equivalent sphere particle, the drug release-rate profiles were similar. This indicates that the single particle technique can be used to compare the dissolution of particles of different particle shape. The simulated dissolution rate−inflow rate relationship bent slightly but the relationship was nearly linear in the range of inflow rates used in the single particle dissolution experiments.

### 3.2. Determination of Dissolution Rate

Pharmaceutical preparations contain a large number of particles and multiparticulate dissolution represents a system property. Multiparticulate systems are, however, difficult to model since the fluid flow occurring around each particle is difficult to fully consider [2,16] and the contact area between fluid and particle is based on a single approximation of particle size and shape. In order to have more well-controlled experimental conditions regarding these aspects, i.e., the particle surface area is accurately determined in real-time and the flow of fluid around the dissolving particle is a controlled laminar flow of defined velocity, a single particle dissolution technique represents a more qualified situation.

Using the single particle technique presented in this paper makes it possible to evaluate different fundamental components, and how they affect the dissolution process. For example, various particle sizes and shapes, fluid velocities and dissolution media can be studied in different settings to assess what effect this might have on the IDR. This could assist in a better understanding of in vivo dissolution and to define the most suitable dissolution test conditions. Further, this technique is especially promising for poorly water-soluble compounds as previous studies have pointed out the difficulties of measuring the IDR from standardized dissolution methods, i.e., a compressed disc, since the dissolved material can be below the detection limit [11,32].

In recent decades, dissolution from single particles has been the subject of several studies, allowing the particle size and shape to be accurately observed [2,15,16,17,33,34,35]. The methodology used in these experiments differed, some of which are described here below. Marabi et al. [2] used a microscopy-based experimental method and image analysis algorithms to study sucrose particles (D (v,0.5) = 586 µm) of a spherical shape and homogeneous composition. Prasad et al. [15] used paracetamol single crystals in the size of 3–5 mm grown in an aqueous solution containing 6.02% *w*/*w* p-acetoxyacetanilide (PAA). The paracetamol crystal was glued to a steel needle and positioned appropriately to the direction of the solution flow. Østergaard et al. [34] used lidocaine single crystals (needle-shaped; 2–3.5 mm) obtained from the recrystallization of lidocaine in n-hexane. The single crystal of lidocaine was placed in a dissolution cell and the dissolution studies were performed using UV imaging. Svanbäck et al. [17,35] used an optofluidic flow cell and image analysis to study the dissolution of spherical pellets (14–747 µg) produced from micronized powders. They developed a flow-through cell, wherein the solvent flow did create a centrally positioned particle trapping vortex where the particle was allowed to rotate randomly. A general disadvantage of these methods are the procedures used to prepare the particles suitable for further dissolution investigation.

In this study, a single particle dissolution technique is used which allows original unmanipulated drug particles to be studied, thus avoiding any preparation procedure such as spheronization. The particles used in this study had an initial mass of 0.06–0.88 µg and a calculated initial $D_{p}$ of 37.5–104.6 µm, which are considerably smaller, compared to the methods mentioned above. Even though we acknowledge the fact that a spheronization or a crystallization procedure may give a particle that is easier to study, such preprocessing procedures may change the physical properties of the drug in such a way that it affects the dissolution behavior. Moreover, the simulation discussed above indicates that the shape of the particle affects the local flow pattern of the dissolution fluid around the particle but has a limited effect on the dissolution rate profile.

The micropipette used during the single particle dissolution measurements had a tip of a Ø ≈ 50 µm and the particles selected for the dissolution experiments could not be smaller than about 50 µm. Hence, the particles selected were of similar size and the approximate average size used in the measurements was 115×75×45 µm^3^ for carbamazepine, 110×50×30 µm^3^ for ibuprofen and 95×50×20 µm^3^ indomethacin (L×B×T). During the experiment, one side of the particle was, to a relatively large extent, covered by the tip area of the micropipette which was compensated for in the derived dissolution data (see Equation (5)).

**Figure 9:**
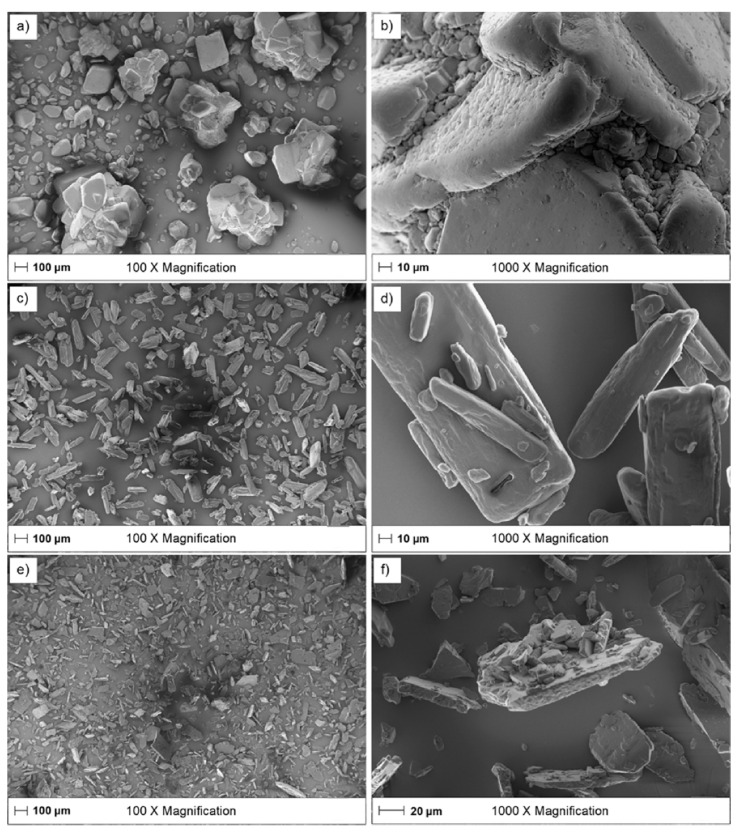
Scanning electron microscopy images of (**a**) carbamazepine 100× magnification, (**b**) carbamazepine 1000× magnification, (**c**) ibuprofen 100× magnification, (**d**) ibuprofen 1000× Magnification, (**e**) indomethacin 100× Magnification, (**f**) indomethacin 1000× Magnification.

The particle shape of the tested compounds varied, i.e., carbamazepine had a more rounded shape (Figure 9a,b) while the shapes of ibuprofen and indomethacin were flat and elongated (Figure 9c–f). The particles did not have any visible open pores, as judged from SEM images, and the particles were considered to be dense. In the case of porous particles, the single particle technique could potentially be used to study the effect of particle porosity on the IDR. For each compound there was also a variation in the main particle dimensions (B, L and T) between each single particle sampled for dissolution measurement, as exemplified for ibuprofen (Table 3). As can also be noticed in the SEM images, the finer particles were adsorbed to the surface of the larger particles (Figure 9b,d,f). These fine particles could not be observed in the microscope used during dissolution testing. It is possible that these adsorbed fine particles can detach from the carrier particle while in contact with a fluid. If this happens when the actual dissolution experiment is started it may give rise to a, in relative terms, fast initial dissolution rate that is not representative of the overall dissolution process. 

**Figure 10:**
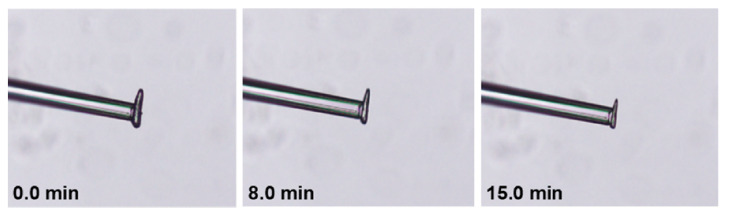
Images of an ibuprofen particle at different time points (0.0, 8.0 and 15.0 min) during an on-going dissolution measurement in the micropipette-assisted microscopy technique.

In Figure 10, the position of an ibuprofen particle on the micropipette is shown. The L, the B and the T of each particle were measured before starting the experiment. Once the particle was attached to the micropipette and the experiment was commenced, the L and the T were measured regularly throughout the experiment. The micropipette kept the particle in a fixed position during the course of the dissolution process, enabling an accurate measurement of L and T. The third particle dimension (B) had to be calculated and to address the question of the accuracy of the calculated estimate of B, the final particle breath of ibuprofen was measured after the experiment was completed for two of the dissolution experiments (46 mm/s ‘measurement 3’ and 66 mm/s ‘measurement 3’). The estimated B values were 28.6 µm and 27.9 µm, while the final measured B values were 30.0 µm and 28.0 µm. It is concluded that the calculation used gave an acceptable approximation of B.

**Figure 11:**
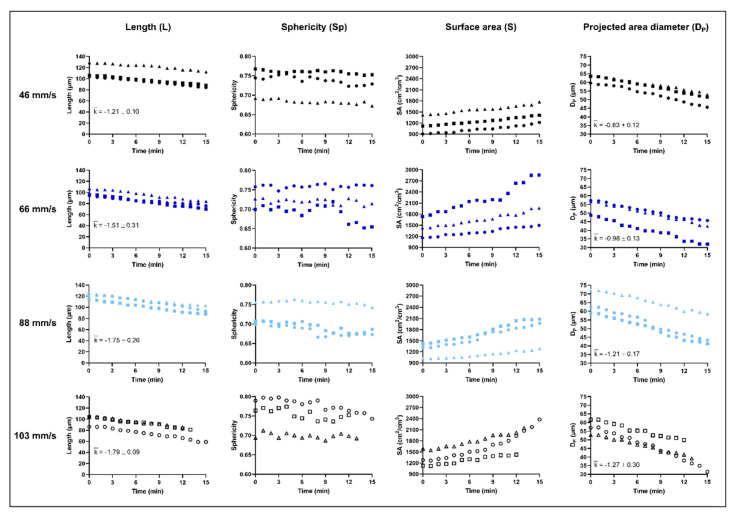
The change in length (L), sphericity (Sp), surface area (S) and projected area diameter ($D_{p}$) of the twelve individual ibuprofen single particles during the IDR measurements using four different fluid velocities; 46 mm/s, 66 mm/s, 88 mm/s and 103 mm/s.

The data collected during a dissolution experiment are exemplified (Table 3) for one of the compounds used, i.e., ibuprofen. The initial mass of the sampled ibuprofen particles varied between 0.07 µg and 0.28 µg and the initial $D_{p}$ between 48.8 µm and 71.9 µm. The particle surface area ($S_{0}$) flakiness (F), and sphericity (Sp) also varied between the single particles. The dissolution measurement was performed as long as possible with the experimental set-up used. For ibuprofen, this occurred typically after about 15 min when the particle either detached, rearranged on the micropipette or the particle was broken into two pieces. For the dissolution measurement to be feasible, the particle has to be attached and retained on the micropipette for a sufficient time-period. For example, if the particle detached too soon, the measurements had to be restarted. By comparing the initial particle (0 min) to the same particle after 8 and 15 min of dissolution (Figure 10) an obvious decrease of both the L and T of the particle was seen. From the measurements of L and T, the change in other indications of particle dimensions were derived and in Figure 11, the change in L with dissolution time for ibuprofen is presented together with three other indications, i.e., Sp, S and $D_{p}$. Results are presented for the individual particles measured for each fluid velocity.

The L and the $D_{p}$ of the particles decreased throughout the experiments approximately linearly with time and the decrease became more rapid with a higher fluid velocity, i.e., the average slope of the line ($\bar{k}$), calculated by linear regression, increased with an increase in flow velocity. The absolute surface area of the particle decreased with a reduction in particle diameter but the specific surface area increased during the dissolution process. The specific surface area profiles were nearly linear but tended to bend upwards with increased dissolution time. 

The sphericity (Figure 11) decreased slightly with dissolution time and the flakiness (i.e., L/T) changed from a range of 2.7 and 5.0 before dissolution to a range between 3.4 and 5.8 as the dissolution measurements proceeded. It seems, therefore, that the particles became flakier as the dissolution measurement proceeded in the set-up used. One possible explanation is that the side of the particle facing the incoming fluid dissolved faster. This is consistent with the CFD simulations (Figure 6) where a thicker stagnant layer was seen on the part of the particle that was not facing the incoming flow. However, compared to the change in L and $D_{p}$ of the particles, the change in particle shape was small and thus, the particle shape was almost retained during the course of dissolution.

**Figure 12:**
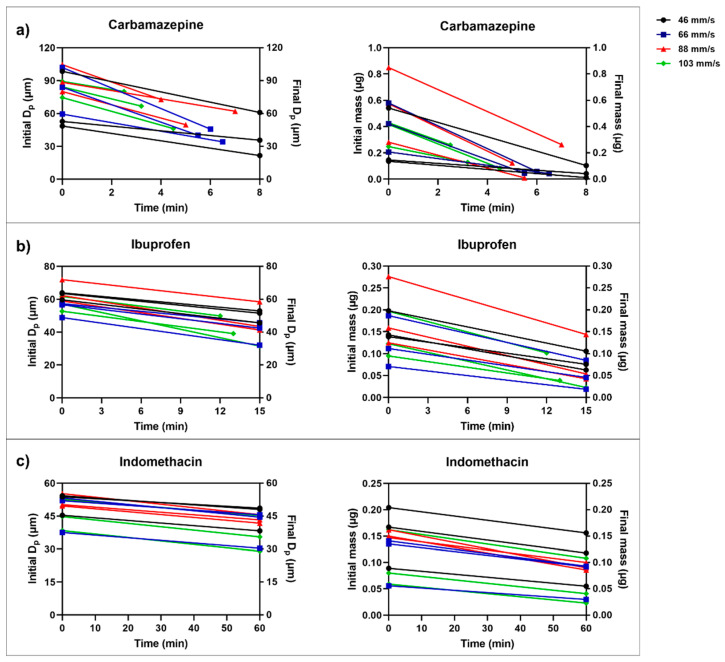
The initial and final $D_{p}$ and mass of (**a**) carbamazepine, (**b**) ibuprofen and (**c**) indomethacin single particles during each individual dissolution measurement using different flow velocities (46–103 mm/s).

The highest fluid flow velocity used in this study was 103 mm/s. At this flow velocity, some of the particles detached from the micropipette when the flowing fluid came in contact with the particle, i.e., the suction applied was not always strong enough to hold the particle firmly attached to the micropipette. It is thus also possible that a tendency for a reorientation of the particle at the tip of the pipette during the course of dissolution increased at the highest flow velocity. For the same reason, the minimum final $D_{p}$ of carbamazepine, ibuprofen and indomethacin that could be determined was approximately 30 µm (Figure 12), since below this particle size, the particles became too small to be firmly attached to the micropipette. If there is a need to measure dissolution of even smaller particles, i.e., a $D_{p}$ below 30 µm, a smaller tip diameter would need to be used. Since the measurement proceeded until this limit of $D_{p}$, the measurement time varied between the compounds, depending on their solubility (Figure 12). For carbamazepine, the single particles dissolved relatively rapidly, i.e., in about 4–5 min, while for indomethacin, the particle dissolution measurements were maintained for 60 min in all flow velocities. 

One of the compounds used in this study, carbamazepine, is reported to have several different anhydrous polymorphs and a dihydrate form, which exhibit different melting points, solubility and compactability [20]. The most common forms of carbamazepine are the anhydrous form III and the dihydrate form. The anhydrous form III is thermodynamically stable at room temperature, while carbamazepine dihydrate is the most stable form in aqueous solution. Hence, all polymorphs convert to the dihydrate form in aqueous solution through a solution-mediated mechanism [36].

In a previous study where disc IDR was performed [20], it was shown that particle size and morphology affected the appearance of the surface of the compacts. To minimize this problem, carbamazepine samples were sieved into fractions and a size fraction of 250–355 µm was selected. When measuring IDR, the slop of the curve changed because of a phase transformation from anhydrous into the dihydrate form. During the IDR measurement, crystallization of carbamazepine dihydrate occurred at the surface of the disc, resulting in a decrease of IDR and in two distinct slopes. The initial slope represented the dissolution of the anhydrous phase and the second slope described the dissolution of the dihydrate phase. In another study [37], where disc dissolution of poorly soluble weak acids was investigated, a similar morphological transformation was observed during the experiment. As one example, naproxen exhibited surface heterogeneity to start with, but the appearance of the surface changed during dissolution, where hydrate formation was suggested as an explanation.

**Figure 13:**
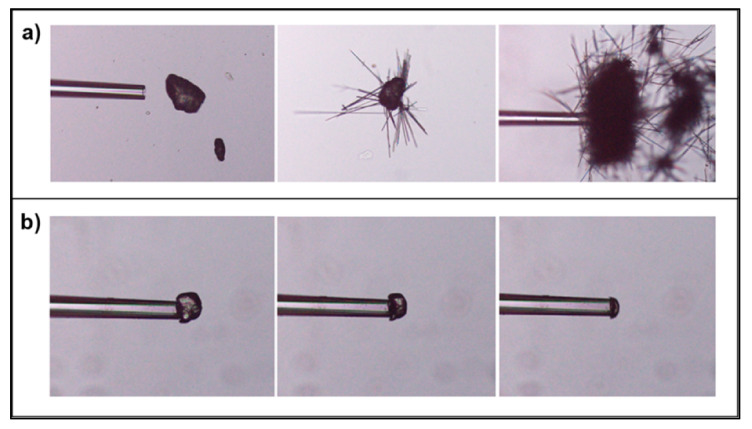
(**a**) A single particle of carbamazepine converting to a dihydrate form in aqueous solution where a needle shaped form occurs (no fluid flow), (**b**) A single particle dissolution experiment of carbamazepine under controlled fluid conditions (66 mm/s) where no transformation took place.

In this study, a change in the appearance of the particles was observed while held in water, giving the formation of a large number of small aggregated needles (Figure 13a). This indicates a phase transformation into the dihydrate form of carbamazepine. However, during single particle dissolution in a continuous aqueous fluid flow, a similar change in the appearance of the studied particle was not observed (Figure 13b). Thus, the phase transformation did not occur during the actual dissolution measurements. The type of single particle dissolution technique used in this study, with a continuous monitoring of the particle appearance, could be a complementary technique to other dissolution experiments to detect phase transformations occurring during dissolution and determine any consequent effect on the dissolution rate. 

### 3.3. Intrinsic Dissolution Rate

From the dissolution data for single particles of carbamazepine, ibuprofen and indomethacin, a surface area normalized dissolution rate (SAND), hereafter denoted IDR, was derived at four fluid velocities. The IDR values were calculated firstly, by Equation (9) and secondly, as the slope of the plot of the cumulative dissolved amount of compound per surface area (µg/cm^2^) against time (min). The latter procedure gave lower standard deviations of IDR in all cases except for ibuprofen 103 mm/s and indomethacin 46 mm/s and 103 mm/s (Table 4). 

The average relative standard deviations (%RSD) of the dissolution measurements were 8.4% for carbamazepine, 12.8% for ibuprofen and 14.5% for indomethacin, comparable to a %RSD of 9.4 reported by Svanbäck et al. [17]. The measurements performed in the highest fluid velocity (103 mm/s) gave the highest variability in dissolution data, explained by a tendency to a reorientation of the particle at the tip of the pipette during the course of dissolution, as discussed above. 

**Figure 14:**
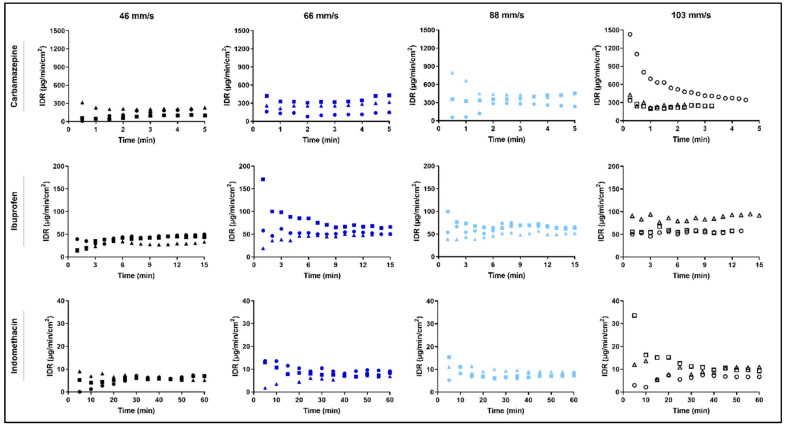
Intrinsic dissolution rate (µg/min/cm^2^) of the single particle dissolution measurements plotted against time for carbamazepine, ibuprofen and indomethacin, here presented separately for each fluid velocity, 46 mm/s, 66 mm/s, 88 mm/s and 103 mm/s.

The IDR was relatively constant during the dissolution process (Figure 14) and the variations obtained thus seemed stochastic with one exception, i.e., relatively high IDR values were obtained during the first minutes (about 3 min) of dissolution in some cases. These initial high values may be due to the detachment of small drug particles attached to the surface of the particle (Figure 9) and thus affecting the measurement of L and T, as discussed above. An alternative explanation is that a slight reorientation of the particle occurred when the dissolution experiment started. Another explanation is that the IDR values calculated by Equation (9) fluctuate more in the beginning as the distance between the data points are narrow which gives rise to more uncertain data. As the distance between the initial $W_{0}$/$S_{0}$ and the data points collected at time ‘t’ increases, the fluctuation eventually evens out. 

**Figure 15:**
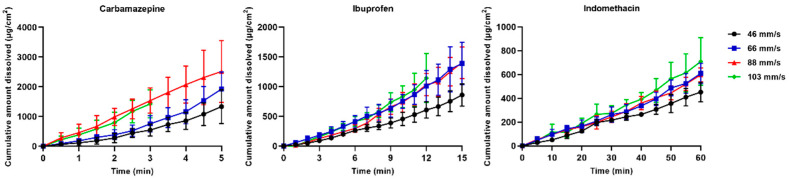
The cumulative amount of dissolved drug (µg/cm^2^) presented as mean ± SD in each fluid velocity (46–103 mm/s) for carbamazepine, ibuprofen and indomethacin.

The cumulative amount of dissolved drug (µg/cm^2^) was plotted against time (min) to demonstrate the dissolution process of the single particles (Figure 15). The particles dissolved in a nearly linear way, and with an increase in dissolved amount per time with an increase in fluid velocity. However, as mentioned earlier, one limitation was the difficulty in measuring the dissolution in the highest fluid velocity (103 mm/s). For example, carbamazepine could be measured for only a few minutes, which made the results somewhat uncertain in this fluid velocity. The most poorly soluble drug, indomethacin, could be measured for a time period of 60 min in all fluid velocities. 

**Figure 16:**
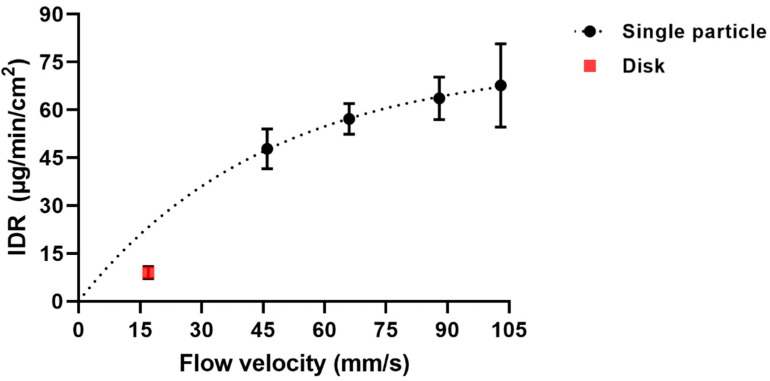
Intrinsic dissolution rate (µg/min/cm^2^) as a function of flow velocity (mm/s) for ibuprofen single particles (black circles ●) and a disc dissolution measurement (red square ■). The dotted line is hypothetically drawn to illustrate a possible relationship between a disc dissolution measurement (flow velocity 17 mm/s) and single particle dissolution measurements (flow velocity 46–103 mm/s). This figure can be compared to Figure 8 showing a similar relationship.

The IDR increased with an increase in fluid flow velocity in a slightly bent way (Figure 16). In the same range of flow velocity, the simulations gave a similar type of relationship between Sherwood number and flow velocity (Figure 8), supporting that a controlled laminar flow occurred during the dissolution experiment. For ibuprofen, an IDR was also determined by a rotating disc method using a µDISS profiler. The IDR thus obtained was 9.0 ± 1.9 µg/min/cm^2^, which was markedly lower than the IDR obtained by the single particle dissolution technique. The fluid flow velocity in the µDISS Profiler using a disc stirrer has been estimated to be [28] about 17 mm/s which is considerably lower than for the single particle technique used here, i.e., 46–103 mm/s. Assuming that the type of bent relationship between IDR and flow velocity obtained by simulation (Figure 8) applies also to the experimental relationship, the IDR obtained by the disc method is somewhat lower than the extrapolated single particle results. This may indicate that the fluid flow conditions in the vicinity of the rotating disc is less well defined than in the single particle set-up used in this study. As a future study, it would be interesting to compare the results obtained in this study to dissolution data obtained by an USP 4 dissolution apparatus which has a similar fluid flow pattern, provided that an accurate solid-to-liquid contact area can be determined for a polydisperse powder.

**Figure 17:**
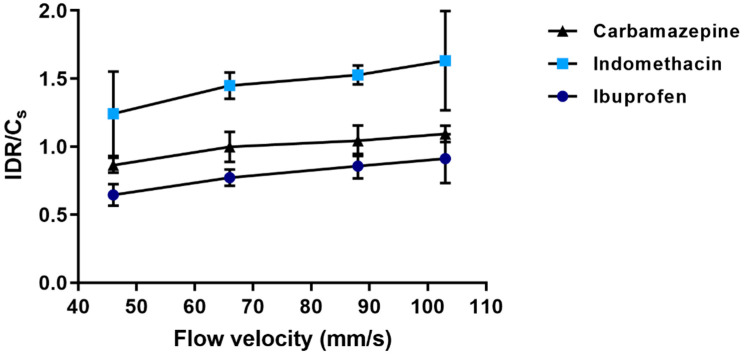
The ratio of IDR and solubility ($C_{s}$) (presented as mean ± SD) as a function of flow velocity (mm/s) for carbamazepine (black triangle ▲), ibuprofen (light blue squares ■) and indomethacin (blue circles ●).

The IDR increased with an increased solubility of the compounds, albeit not in a linear way. Since laminar flow occurred around the particle, an effective transport rate constant describing the transport of dissolved molecules in the stagnant water layer can be calculated as the ratio between IDR and solubility (Figure 17). The qualified measurement conditions used in this study make it possible to decipher the dissolution curve into two physically sound descriptors of the dissolution process, i.e., the IDR and effective transport rate constant. Hence, the relative contribution of drug solubility and drug transport properties on the IDR can be assessed. The high IDR of carbamazepine is a consequence of both a high solubility and a high transport rate, while the relatively low IDR of ibuprofen in relationship to the solubility is due to a low transport rate. 

The effective transport rate constant increased for all compounds nearly linearly with flow velocity in the range of velocities used, reflecting a decreased thickness of the aqueous boundary layer and hence, a reduced effective diffusion distance of the solute with increased flow velocity.

## 4. Conclusions

In this paper, the intrinsic dissolution rate (IDR) of single particles as well as the solute effective transport rate were determined under laminar fluid flow at a series of fluid velocities. The size of the particles used had a $D_{p}$ of 37.5–104.6 µm and the dissolution was monitored by a micropipette-assisted microscopy technique for periods of 5 to 60 min. 

From CFD simulations, it was concluded that laminar fluid flow occurred around the particles during the dissolution process. The dissolution rates were theoretically investigated for both box shaped and spherical particles and it was found that although differences in local dissolution behavior could be observed, the overall drug release rate was similar for the box particle and the sphere. Thus, differently shaped particles can be studied by the technique used and the IDR values obtained can be compared. The drug release rate increased non-linearly with fluid velocity.

The experimentally determined IDR increased with fluid velocity in a way which was consistent with the theoretical investigation. The solute effective transport rate increased nearly linearly with fluid velocity due to a decreased thickness of the aqueous boundary layer. The compounds used could be ranked regarding the IDR and effective transport rate of the solute and the influence of the fluid velocity under laminar flow determined.

In summary, qualified single particle dissolution measurements under well-defined conditions gave dissolution data of high quality. To further improve the usefulness of such measurements, calculations of the effective diffusion layer thickness can be done and compared to assessments of the thickness of the aqueous boundary layer by CFD.

## Figures and Tables

**Table 1:** The solubility in water and physicochemical properties of carbamazepine, ibuprofen and indomethacin.

Compound	S_water_ (µg/mL)	Mw ^b^ (g/mol)	logP ^b^	pKa ^b^	Tm ^b^ (°C)	ρ (g/cm^3^)
Carbamazepine	284.9 ± 3.9 ^a^	236	2.8	n	190	1.34
Ibuprofen	74.1 ± 7.1	206	4.0	4.9 (a)	76	1.12
Indomethacin	5.43 ± 0.46	358	4.3	4.5 (a)	151	1.38

^a^ The solubility of carbamazepine in water was extracted from the literature [20]. ^b^ The molecular weight (MW), logP, pKa and melting point (Tm) were collected from the DrugBank database [21].

**Table 2:** Parameter values used in the numerical simulation.

Quantity	Symbol	Value	Unit
Fluid density	$\rho_{f}$	998.2	$\mathrm{kg}/m^{3}$
Dynamic viscosity	μ	1.0093	mPa⋅s
Diffusion coefficient	D	$5.5\times 10^{-10}$	$m^{2}/s$
Solubility	$c_{s}$	74.1	$\mathrm{mol}/m^{3}$

**Table 3:** Length (L), breath (B), thickness (T), mass, projected area diameter ($D_{p}$), surface area (S), flakiness (F) and sphericity (Sp) of ibuprofen single particles used in each measurement.

Flow Velocity	L (µm)	B (µm)	T (µm)	Mass (µg)	$D_{p}$ (µm)	S (cm^2^/cm^3^)	F	Sp
**46 mm/s**								
- Measurement 1	104	44	28	0.14	59.6	908	3.71	0.74
- Measurement 2	106	56	33	0.20	63.5	1121	3.21	0.77
- Measurement 3	129	37	26	0.13	63.9	1422	4.96	0.69
**66 mm/s**								
- Measurement 1	94	65	30	0.19	57.3	1158	3.13	0.76
- Measurement 2	96	34	22	0.07	48.8	1733	4.36	0.70
- Measurement 3	107	40	27	0.11	56.6	1430	3.96	0.73
**88 mm/s**								
- Measurement 1	124	47	25	0.16	62.2	1325	4.96	0.70
- Measurement 2	114	41	25	0.13	58.9	1419	4.56	0.71
- Measurement 3	122	61	38	0.28	71.9	1020	3.21	0.75
**103 mm/s**								
- Measurement 1	86	43	31	0.12	57.0	1280	2.77	0.79
- Measurement 2	105	59	32	0.20	61.7	1129	3.28	0.76
- Measurement 3	104	39	29	0.10	52.7	1586	4.52	0.69

**Table 4:** Intrinsic dissolution rates (µg/min/cm^2^) of carbamazepine, ibuprofen and indomethacin single particles in four different fluid velocities. IDR were derived using Equation (9) (IDR calculated) or by using the slope of a plot of the cumulative dissolved amount of compound per surface area (µg/cm^2^) against time (min) (IDR slope).

	IDR Slope (µg/min/cm^2^)	IDR Calc. (µg/min/cm^2^)	IDR Slope (µg/min/cm^2^)	IDR Calc. (µg/min/cm^2^)	IDR Slope (µg/min/cm^2^)	IDR Calc. (µg/min/cm^2^)
	Carbamazepine	Carbamazepine	Ibuprofen	Ibuprofen	Indomethacin	Indomethacin
46 mm/s	246.0 ± 15.7	207.8 ± 43.1	47.8 ± 6.2	39.3 ± 6.5	6.74 ± 1.68	6.16 ± 0.36
66 mm/s	284.7 ± 31.3	270.8 ± 88.6	57.2 ± 4.8	55.0 ± 6.9	7.87 ± 0.53	7.82 ± 1.11
88 mm/s	297.3 ± 31.9	342.4 ± 85.0	63.6 ± 6.7	57.9 ± 7.2	8.22 ± 0.33	7.95 ± 0.95
103 mm/s	311.6 ± 17.0	305.5 ± 66.8	67.7 ± 13.1	66.6 ± 7.8	8.86 ± 1.98	8.63 ± 1.29

## Data Availability

Not applicable.

## Abbreviations

$A_{p}$—projected area of a single particle; B—breadth of a single particle; c—drug concentration; $\dot{c}$—material time derivative of the drug concentration c; CFD—computational fluid dynamics; d—inner diameter of the flow-pipette; D—constant diffusion coefficient; $D_{p}$—projected area diameter of a single particle; F—flakiness of a single particle; IDR—intrinsic dissolution rate; K—dissolution rate constant; l—diameter of a tube or a single particle, L—length of a single particle; M—mass of a single particle; P—perimeter of a single particle; Q—volume flow rate; r—radius of the micropipette; Re—Reynolds number, $S_{eff}$—effective interfacial area; Sp—sphericity of a single particle; $S_{0}$—surface area of the initial single particle; $S_{t}$—surface area at time ‘t’ of a single particle; S—surface area of a single particle; $S_{eq}$—surface area of a sphere of equivalent volume to the single particle; SAND—surface area normalized dissolution rate; SEM—scanning electron microscopy; Sh—Sherwood number; T—thickness of a single particle; U—flow speed, v—fluid velocity; $v_{kin}$—kinematic viscosity, $\bar{v}$—average velocity; 2$\bar{v}$—maximum velocity; $\dot{v}$—material time derivative; V—volume of a single particle; $W_{0}$—initial mass of a single particle; $W_{t}$—mass remaining at time ‘t’; μ—dynamic viscosity; $\rho_{p}$—density of a single particle; $\rho_{f}$—fluid density; $\nabla^{2}v$—spatial Laplacian of the fluid velocity; ∇p—spatial pressure gradient; $\phi_{0}$—release rate from a spherical particle of radius.
